# Medical Education in War-Time

**Published:** 1918-09-07

**Authors:** 


### MEDICAL EDUCATION IN WAR-TIME.

During the past twelve months the situation in the teaching hospitals has become far easier owing largely to two factors. The military authorities have at last formulated a definite system of calling up, and the hospitals themselves, knowing exactly how they stand with reference to the Military Service Act, have instituted a system of unqualified house appointments for which senior students are eligible under the guidance of a half or a third the normal number of qualified residents. The students themselves, no longer under any uncertainty as to how they should act (*vide* The Hospital, September 8, 1917, page 456), can now proceed to qualification in a perfectly ordinary and undisturbed fashion.

But it must not be supposed that the National Service authorities are disposed to allow the *ægis* of the medical school to be a pretext for shirking or a haven to the faint-hearted. A careful scrutiny is made of examination lists, and the persistent failure of a student to qualify is apt to be followed by results startling and salutary. There is no place for the "chronic" in war-time. Nor is the pretext of medical studies made an excuse for altogether evading military duties. The O.T.C. holds almost daily drills, and failure to complete a certain number means a polite but authoritative intimation from the War Office. The Navy, too, claims its quota of fourth and fifth year men as surgeon probationers, who serve for a period of six months and are then released to complete their examinations. As yet no system of compulsion has been applied, a requisite number having always been obtained by voluntary means, but in the event of a shortage conscription will be introduced.

### The Difficulties of Medical Schools.

The maintenance of an adequate teaching staff is one of the most difficult problems with which medical schools are at present faced. The specialist has never been in greater demand than to-day, and there is hardly a consultant whose services have not been enlisted for military work. In most hospitals routine surgery and medicine lectures have been abandoned, without—softly be it spoken—any remarkable falling off of the pass lists. But bedside teaching is another matter, and there is no doubt that many a student is severely handicapped for lack of the old-time thoroughness in this respect. The finer points of diagnosis can only be appreciated by actual clinical demonstration, and the average student, who is not an embryo Sydenham or Trousseau, is only too apt to miss many points, unemphasised by the text-books, which he would readily appreciate if pointed out by his teacher. Against this must be set the unrivalled opportunities for practical work of all kinds which the shortage of qualified men affords. The newly qualified man is often called upon to perform urgency operations, and carry out treatment that in other days would have been performed by the head of the department. Clinical material is abundant, and there are few who have not availed themselves of such opportunities. Three months is allowed after qualification for a house appointment, but under the system referred to above, many have already held resident posts for six months before qualifying.

### No Shortage of Doctors.

The number of students actually engaged in medical studies has not shown the falling off that might have been anticipated—indeed, paradoxical as it may seem, there has actually been an increase in the numbers of *men* students in the last two years, whilst, of course, the number of women has increased rapidly. The estimated total of students due to qualify within the next five years is 7,630, of whom 5,380 are men and 2,250 women.

Another notable feature is the compulsorily early age at which studies are commenced, rendered necessary by the fact that a youth on attaining the age of eighteen is called up for service unless within six months of his intermediate examination. We cannot ourselves regard this with unmixed satisfaction. Medical studies require a

**Medical Education in War-Time**—(*continued*).

maturer judgment than is usually possessed by a youth of sixteen, whilst the doctor of twenty-one or thereabouts would not, we fancy, have an extensive following in private practice. Parents will in all probability, however, revert to the more usual age of eighteen or nineteen when normal conditions are resumed. The woman doctor more than holds her own, the London and University College Hospitals being the latest to open their portals to the lady student. That all the large teaching hospitals should not do the like is, we feel, to be regretted. The mere man has much to learn in keenness and application from his women *confrères*, and one can conceive a healthy spirit of rivalry existing between the two, whose influence would be all to the good. Already women have been granted commissions in the R.A.M.C., and it is high time these few relics of sex exclusiveness were swept away.

### Changes in the Medical Curriculum.

There is much evidence to show that the existing system of medical education will in the near future be subjected to thorough revision. The increasing scope of preventive medicine, the elaboration of exact and highly technical methods of diagnosis and treatment, can mean but one thing—that the private practitioner as such must give way to a specialised State Service, or that some means must be devised to keep medical training abreast of the times, and to familiarise the student with the latest methods of laboratory technique and advances in therapeutics. The day when medical treatment was confined to a bottle of medicine and a little elementary hygienic advice are past. The vast domains of bacteriology, massage, radiography, electro-therapeutics and the rest cannot be passed over in silence if the pioneer work of the last quarter of a century is to be of permanent benefit to the community at large. The interesting suggestions put forward by Sir George Newman (see The Hospital, August 17, page 423) have already been dealt with in these columns. But while we fully admit the great value of preliminary science studies and concur with his proposal that much of this elementary teaching could be dealt with by the secondary schools, yet we cannot but feel that by too great insistence on the value of a scientific training we are losing sight of the prime function of the work-a-day physician—which is to heal.

### The Doctor as Scientist.

That every doctor should be a man of science is, doubtless, an ideal to be sought after. That under existing conditions it is in any way attainable we cannot believe. The present curriculum—largely, perhaps, owing to the system of watertight compartments, by which, for example, physiology is divorced from clinical medicine, pharmacology taught before the student has ever seen a patient in the wards, and botany remains as a curious survival of the days when the doctor culled his herbs from the hedgerow—means simply that the student's brain is filled with a plethora of isolated and ill-digested information which he contrives to forget with all speed the moment the need for it has passed. The more esteemed the degree, the more does our present examinational system reflect the bias of the professorial mind. What can we say of a system which demands a quite extensive knowledge of embryology for its Intermediate examination, but in the Finals passes the living infant by, with all its complexities of nutritional disorders, as unworthy of the academic mind—a system which considers the relationship of xylem and phloem to be more essential to the future physician than a course of normal psychology? It is needless to multiply such anomalies. A glance at the curriculum required for any university course will furnish many instances equally striking. The truth of the matter is that, as things stand at present, the routine practice of medicine does not yield enough to warrant a student spending ten years or more upon his professional studies. That being so, we must obviously lighten the course of much of its purely academic baggage. That six months of a five and a half years' course should be devoted to the study of organic chemistry (including, for example, the commercial methods of manufacture of compounds common and uncommon), whilst the whole subject of midwifery and gynæcology is disposed of in half the time, is a piece of gratuitous pedantry that none but a board of university professors could have perpetrated. One does not wish to decry the value of knowing the precise method by which citric acid is obtained from Messina lemons, but we submit that a course of metallurgy, in order to be conversant with the manufacture of steel for scalpels, would be of equal value for the practising physician.

### The Human Side.

Some words of Professor Haldane* seem so apposite that we cannot refrain from quoting them: "A human being is far more than the mere organism with which medicine and surgery are immediately concerned. To a good doctor his patients, as fellow-men, have a value which cannot be measured; and he has constantly to deal with them on the human side and adapt his treatment to it. If he fails to do so, he emphatically fails in his duty. In thinking of the scientific side of medical education we are sometimes apt to let slip the human side, and forget that an essential part of the medical education consists of those subtle influences, exercised mainly by example, though partly also by special instruction, which teach sympathy with and understanding of human personality. If, therefore, medicine is to have more science and less rule of thumb, let us see to it also that with the science goes more humanity, and, where possible, a broader general education, not in the mere dry bones of the humanities, but on living, humanistic lines."

* J. S. Haldane, "The Relation of Physiology to Medicine," *Edinburgh Med. Journ.*, April, 1918.

